# Refining Lineage Classification and Updated RFLP Patterns of PRRSV-2 Revealed Viral Spatiotemporal Distribution Characteristics in China in 1991–2023

**DOI:** 10.1155/tbed/9977088

**Published:** 2025-03-09

**Authors:** Xiaoxiao Tian, Ziyi Wei, Mirwaise Khan, Zhi Zhou, Jianqiang Zhang, Xinyi Huang, Yongbo Yang, Shujie Wang, Haiwei Wang, Xuehui Cai, Fandan Meng, Tongqing An

**Affiliations:** ^1^State Key Laboratory for Animal Disease Control and Prevention, Harbin Veterinary Research Institute, Chinese Academy of Agricultural Sciences, Harbin 150069, China; ^2^WOAH Porcine Reproductive and Respiratory Syndrome Reference Laboratory, China Animal Disease Control Center, Beijing 100125, China; ^3^Veterinary Diagnostic and Production Animal Medicine, College of Veterinary Medicine, Iowa State University, Ames, Iowa, USA; ^4^Collaborative Laboratory of Porcine Reproductive Dysfunction Disease for China Animal Disease Control Center, Harbin Veterinary Research Institute, Chinese Academy of Agricultural Sciences, Harbin 150069, China

**Keywords:** genetic diversity, lineage, PRRSV, RFLP pattern, spatiotemporal distribution

## Abstract

Porcine reproductive and respiratory syndrome virus (PRRSV) is a significant infectious disease impacting the global swine industry. Due to high frequency of viral mutation and recombination, PRRSV exhibits complex genetic diversity; however, its lineage classification, restriction fragment length polymorphism (RFLP) patterns, and spatiotemporal distribution have not been systematically analyzed in China. In this study, we sequenced PRRSV-2 open reading frame (ORF)5 sequences from clinical samples (*n* = 364) and retrieved all the available PRRSV-2 ORF5 sequences in China in 1991–2023 from GenBank (*n* = 5773). Systematically analysis revealed that PRRSV-2 strains in China were classified into five lineages (L1, L3, L5, L8, and L9) and eight sublineages (L1A-L1C, L5A, L5B, L8C, L8E, and L9B), the L8E and L1C PRRSV-2 were widely distributed across almost all provinces in China, the L1C and L1A strains were increasing and gradually replacing L8 as dominant epidemic strains, and L1B PRRSV-2 in China was analyzed for the first time. The L3 PRRSV-2 has a trend of spreading gradually from the southern to the northern provinces, which needs to be paid attention to the monitoring and prevention of PRRSV-2. Meanwhile, PRRSV-2 strains in China were classified into 112 different RFLP patterns. RFLP 1-4-4 PRRSVs were detectable in China, which accounted for 12.71% of all Chinese PRRSV-2 strains. Although they are different from the RFLP 1-4-4 L1C variant in the United States, it is necessary to enhance surveillance of the RFLP 1-4-4 L1C PRRSVs. These results contributed the understanding of genetic diversity and spatiotemporal distribution of PRRSV-2 in China and provide important references for future PRRSV-2 monitoring and control in China.

## 1. Introduction

Porcine reproductive and respiratory syndrome (PRRS) is a significant infectious disease that causes reproductive failure in sows and severe respiratory disorders in pigs of all ages, leading to substantial economic losses in porcine industry [1, 2]. The causative agent, PRRS virus (PRRSV), is a single-stranded RNA virus of the genus *Betaarterivirus*, family Arteriviridae, and order Nidovirales [3]. PRRSV comprises two species: *Betaarterivirus suid* 1 (PRRSV-1, previously known as European type) and *Betaarterivirus suid* 2 (PRRSV-2, previously known as North American type), which share 60% nucleotide (nt) identity at the genome level [4, 5]. The PRRSV genome is approximately 15 kb in length and contains at least 10 open reading frames (ORF), including ORF1a, ORF1b, ORF2a, ORF2b, ORF3 to ORF7, and ORF5a [6]. Due to the high heterogeneity of ORF5 gene, it is extensively used to study the genetic diversity of PRRSV [7–9].

Restriction fragment length polymorphism (RFLP) typing was first introduced in 1998 to differentiate the Ingelvac PRRS modified live vaccine (MLV) from wild-type PRRSV strains, using three restriction enzyme sites (*Mlu* I, *Hinc* II, and *Sac* II) located in the ORF5 gene [10]. RFLP typing is easily understood by veterinarians and producers. In the USA, at least 228 distinct RFLP patterns have been reported, reflecting the complex genetic diversity of PRRSV-2 [11]. However, RFLP typing has recognized shortcoming, mutations at the enzyme cutting sites can alter the RFLP pattern [12], which may not accurately reflect genetic relationship among PRRSV-2 strains [13]. To address this limitation, lineage classification was proposed in 2010 to describe PRRSV-2 genetic diversity based on global ORF5 sequences and it has been widely used since then [14]. Due to the continuous mutation of PRRSV-2, new lineages or sublineages PRRSVs constantly emerged [15, 16]. In 2023, the PRRSV-2 lineage classification was refined, updating from 9 to 11 lineages [13]. However, PRRSV-2 sequences in China have not been thoroughly analyzed using the new classification system, and it is unknown if new lineage or sublineage PRRSV has emerged as well as the distribution and prevalence of each lineage in China.

This study aimed to achieve the following objectives: (i) to classify all the available PRRSV-2 ORF5 sequences in China according to the new PRRSV-2 classification system, (ii) to analyze the geographic distributions and temporal changes of PRRSV-2 sequences in China during 1991–2023, and (iii) to compare phylogenetic classification and RFLP typing to better understand the epidemiology of PRRSV-2 in China. The results will contribute to a better understanding the epidemiological dynamics of PRRSV-2 in China.

## 2. Materials and Methods

### 2.1. Clinical Sample Collection

From 2020 to 2023, a total of 659 clinical samples including lungs, spleens, lymph nodes, hearts, and nasal swabs were collected from different pig farms in nine provinces (Heilongjiang, Jiangsu, Shandong, Xinjiang, Fujian, Guangdong, Shanxi, Hunan, and Inner Mongolia) of China. All samples were stored at −80°C until further processing.

### 2.2. Detection and Sequencing of PRRSV-2

The tissue samples were divided into pieces (~0.1 g) and homogenized in phosphate-buffered saline (PBS). From this homogenate, 140 μL supernatant was extracted for total RNA using QIAamp viral RNA Mini Kit (QIAGEN, Germany), and the RNA samples were analyzed by reverse transcription polymerase chain reaction (RT-PCR), and the ORF5 gene of positive samples was sequenced using Sanger method, according to previously described method [17].

### 2.3. Dataset of PRRSV-2 ORF5 Sequences

**Figure 1:**
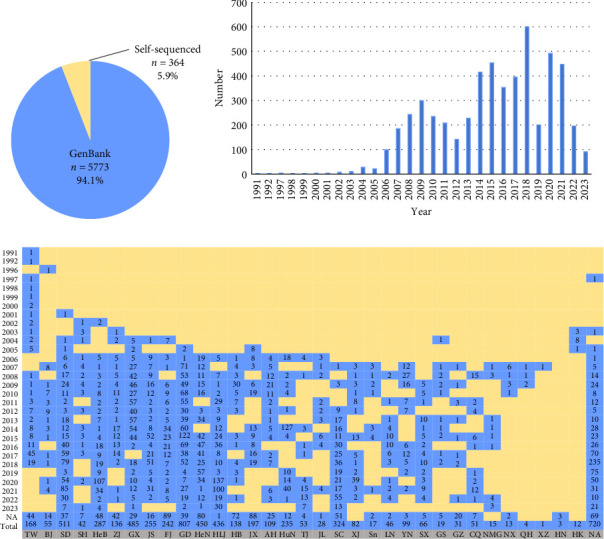
Spatiotemporal distribution of porcine reproductive and respiratory syndrome virus (PRRSV)-2 in China during 1991–2023. (A) The proportion of PRRSV-2 sequenced by our laboratory. A dataset of 6137 PRRSV-2 open reading frame (ORF)-5 sequences was analyzed in this study, 5.9% (*n* = 364) was detected in this laboratory. (B) Temporal dynamics of PRRSV-2 in China. (C) Geographical distribution of PRRSV-2 in China.

This study includes PRRSV-2 ORF5 sequences (*n* = 108) from clinical samples collected in 2020–2023, as well as sequences (*n* = 256) from samples collected in 2013–2020 in our laboratory; furthermore, all available PRRSV-2 ORF5 sequences (*n* = 5773) in China were downloaded from GenBank until November 30, 2023, thus, a merged dataset contained 6137 PRRSV-2 ORF5 sequences was installed (Figure 1A). The background information of 6137 PRRSV-2 strains including GenBank accession number, isolate name, collection year, and location was investigated shown in Supporting Information: Table S1. The 6137 PRRSV-2 sequences were distributed across almost all provinces of China, and the major pig-raising provinces had more PRRSV-2 strains.

### 2.4. Phylogenetic Analyses of PRRSV-2 ORF5 Sequences

Phylogenetic analyses and lineage classification of PRRSV-2 ORF5 sequences were conducted following the methodology described by Yim-im et al. [13]. Multiple sequence alignments were generated using MAFFT7, the maximum likelihood (ML) trees were constructed using iqtree-2.2.2.6 with SYM + ASC + R10 model and 1000 bootstrap replicates [18]. The lineages of PRRSV-2 in China were reclassified according to the 11-lineages classification criteria [13]. To illustrate lineage diversity, representative strains from each lineage were selected and their ORF5 sequences were used to construct a phylogenetic tree. For a detailed analysis of the mutation characteristics of the GP5 protein among different sub-lineages of lineage 1, the dominant lineage circulated in both China and the USA, the reference sequences of sublineages L1A–L1F and L1H–L1J were analyzed using MegAlign software (DNASTAR Inc).

### 2.5. RFLP Pattern Analyses

The RFLP patterns of Chinese PRRSV-2 ORF5 sequences were analyzed. Specifically, 4367 out of 6137 Chinese PRRSV-2 ORF5 sequences had been analyzed in the study of Yim-im et al. [13], so we analyzed the rest 1770 sequences according to the method of Yim-im et al. [13]. All the RFLP pattern results of Chinese PRRSV-2 ORF5 sequences were integrated for subsequent analyses.

## 3. Results

### 3.1. RT-PCR Survey of Clinical Samples

Among the 659 clinical samples analyzed, PRRSV-2 was detected in 108 samples, with a positive rate of 16.39%. Those positive samples were collected from six provinces including Heilongjiang, Shandong, Jiangsu, Guangdong, Inner Mongolia, and Xinjiang. The ORF5 gene of each positive sample was sequenced and all of which were 603 bp in length.

### 3.2. Characteristics of the PRRSV-2 Dataset

In the dataset of 6137 PRRSV-2 ORF5 sequences, the earliest recorded sequence was MD001 strain, which was collected in Taiwan in 1991. Between 1991 and 2005, PRRSV-2 was mainly distributed in Fujian, Jiangxi, Guangxi, Shanghai, Taiwan, and Hong Kong, altogether 74 ORF5 sequences of PRRSV-2 were collected. However, in 2006, a highly pathogenic PRRSV (HP-PRRSV) outbreak in swine herds in Jiangxi province and quickly spread to most provinces in China. Consequently, the number of PRRSV-2 ORF5 sequences increased rapidly from 2006 to 2010. However, with the use of HP-PRRSV live vaccines in 2011, HP-PRRSV has been effectively controlled in China; the number of PRRSV-2 ORF5 sequences has a downward trend from 2011 until 2013 (Figure 1B). Furthermore, along with the emergence of NADC30-like PRRSV in China, the number of PRRSV-2 ORF5 sequences increased. The provinces with a higher number of pigs also have a higher quantity of PRRSV-2 ORF5 sequences (Figure 1C).

### 3.3. Refining Lineage Classification of PRRSV-2 in China

**Figure 2:**
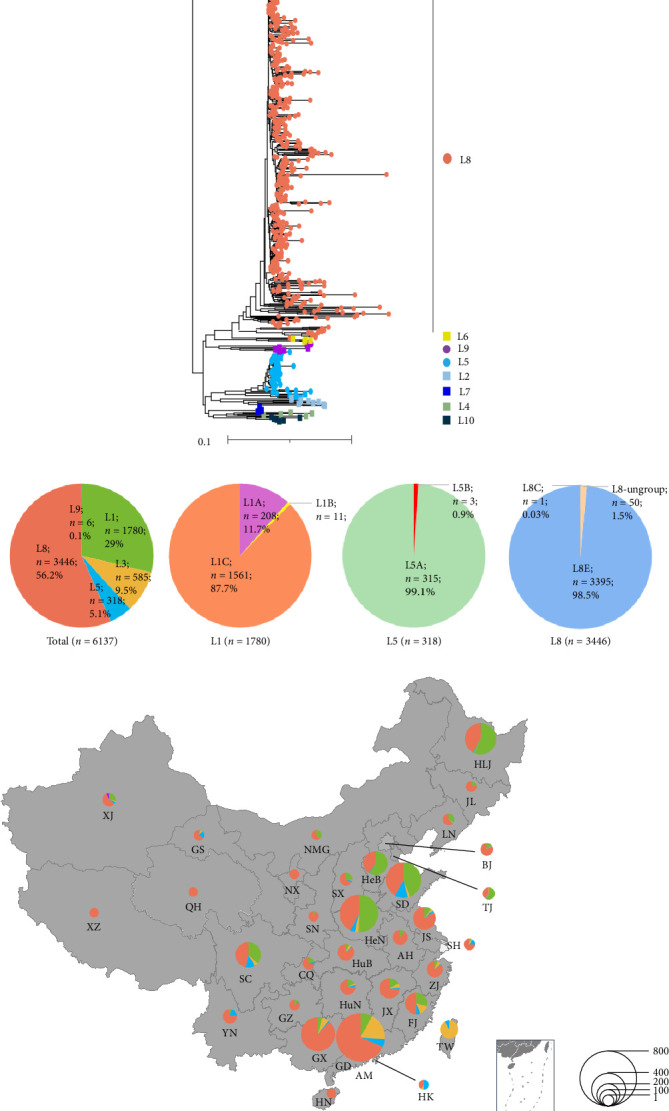
Lineages classification and spatiotemporal distribution of porcine reproductive and respiratory syndrome virus (PRRSV)-2 during 1991–2023. (A) Classification of PRRSV-2 in China during 1991–2023. Based on the newly refined PRRSV-2 classification systems, PRRSV-2 in China were divided into five lineages including L1, L3, L5, L8, and L9. The sphere represents the lineage that exists in China, and the square represents the lineage that does not exist in China but exists abroad. (B) The sublineages of PRRSV-2 L1, L5, and L8 in China. (C) The distribution of PRRSV-2 lineage in China during 1991–2023. The number of sequences and its proportions are illustrated by pie charts in each province. (D) Temporal dynamics of PRRSV-2 lineages in China during 1991–2023. The *y*-axis represents the percentage of lineages detected in different years.

Based on the last 11-lineages classification method of PRRSV-2 [13], the 6137 PRRSV-2 ORF5 sequences were classified into five lineages (Figure 2A), including L1 (*n* = 1780, 29%), L3 (*n* = 585, 9.53%), L5 (*n* = 318, 5.18%), L8 (*n* = 3446, 56.15%), and L9 (*n* = 6, 0.1%). Additionally, 2 out of 6137 PRRSV-2 ORF5 sequences (GenBank No. OL546227 and OL546253) did not belong to any of the 11 lineages (Figure 2B). A total of 1780 PRRSV-2 sequences in L1 were further classified into L1A, L1B, and L1C sublineages, while L1D–L1F and L1H–L1J were not found in China. Most of the L1 lineage PRRSV were L1A (NADC34-like PRRSVs, *n* = 208) and L1C (NADC30-like PRRSVs, *n* = 1561), only 11 sequences were annotated to L1B, which were detected from Shandong, Henan, Anhui, and Shanxi provinces in 1994–2021 (Figure 2B). All the 318 L5 PRRSVs were further classified into L5A (*n* = 315) and L5B (*n* = 3; Figure 2B). In the L8 lineage, all the 3446 ORF5 sequences were further divided into three sublineages, one PRRSV sequence (GenBank No. JQ798258) belonged to L8C sublineage; 3395 PRRSV sequences (CH-1a-like PRRSVs and HP-PRRSVs) belonged to L8E sublineage; 50 PRRSV-2 ORF5 sequences of L8 did not belong to any of the L8 sublineages (Figure 2B). All the L9 PRRSVs in China were all divided into L9B (*n* = 6).

### 3.4. Spatiotemporal Distribution of Different PRRSV-2 Lineages in China

The geographic distribution of PRRSV-2 in China was analyzed based on available collection date and location. At the lineage level, L1, L5, and L8 PRRSVs were detectable in most provinces (Figure 2C). For example, L8 PRRSVs were distributed in all provinces except Taiwan, and the L8 sequences account for a large proportion in some provinces, such as 89.91% in Anhui, 87.84% in Guangxi, and 85.51% in Hubei. For L5 PRRSVs, those were distributed in 26 provinces, but the number of strains in each province was small (less than 63). L1 PRRSVs can be detectable in all provinces except Hainan, Ningxia, Qinghai, and Tibet, with Henan showing the highest detection rate at 16.35%. L3 sequences were mainly distributed in southern coastal areas, such as Fujian, Guangdong, Guangxi, and Taiwan provinces. L9 sequences were only distributed in Xinjiang province. Regarding temporal dynamics, L3 PRRSV was first detected in China in 1991, followed by L5 PRRSV in 1992, and L8 PRRSV in 1996; both L1 and L9 PRRSV were first detected in China in 2011 (Figure 2D). The proportion of L1 sequences increased from 0.49% in 2011 to 71.20% in 2020, meantime the L8 sequences decreased from 84.95% to 21.91%. L1 PRRSV has become the dominant epidemic lineage in China since 2017.

### 3.5. Characteristic Mutations Among Different L1 Sublineages

**Figure 3:**
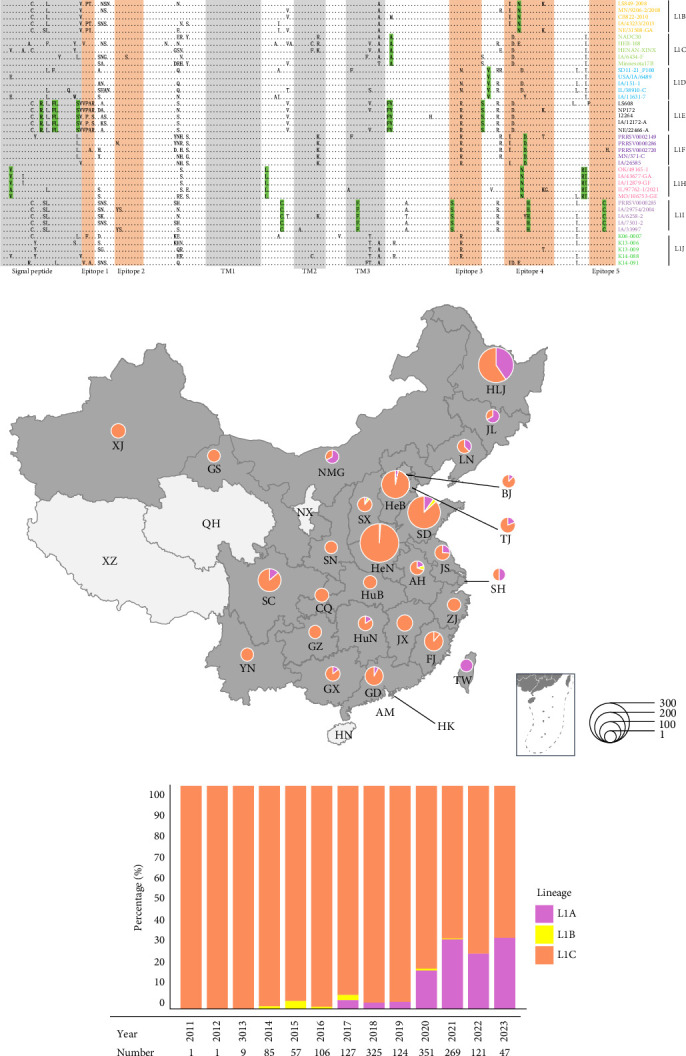
Comparison of amino acid mutations and spatiotemporal distribution of porcine reproductive and respiratory syndrome virus (PRRSV)-2 L1 open reading frame (ORF)-5 sequences. (A) Analysis and comparison of amino acid mutations in PRRSV-2 L1 GP5 protein. To investigate the amino acid difference among PRRSV-2 L1 GP5 protein, the representative strain of L1A, L1B, and L1C in China, together with some strains represented L1D–L1F and L1H–L1J from other countries were aligned. Critical amino acid variations were marked by green color. (B) Distribution of the sublineages in L1 during 1991–2023. The number of strains and their relative proportions are illustrated in each province. (C) Temporal dynamics of PRRSV-2 L1 sublineages in China during 1991–2023.

To investigate the amino acid mutations among sublineages of L1 PRRSV-2 GP5 sequences, representative strains of L1A~L1F and L1H~L1J were aligned. The results showed that the amino acid homology of different sublineages of L1 ranged from 83.1% to 97% (Supporting Information: Table S2). The amino acid mutations were distributed in some motifs of GP5, such as the signal peptide, transmembrane regions, and epitopes. Some sublineage-specific amino acid mutations were identified, the PRRSVs in L1A~L1D have sublineage-specific mutations at positions 98 (T98A), 170 (G170N), 128 (T128A), and 160 (I160V), respectively (Figure 3A). L1F has a sublineage-specific mutation at position 172 (H172D). L1E has seven sublineage-specific mutations, four of which are located in the signal peptide. L1H and L1I each have five sublineage-specific mutations, and three mutations of L1I are located in epitopes ^149^LDTKGRLYRMR^156^, ^164^GGKVEVEGHLIDLKRVV^180^ and ^192^VSAEQMGRL^200^. L1J has no sublineage-specific mutation. The distribution of PRRSV L1 in China was further analyzed. PRRSV L1 was distributed in most provinces of China, among which Henan (*n* = 290, 16.34%) had the largest quantity, followed by Heilongjiang (*n* = 249, 14.03%) and Shandong (*n* = 226, 12.73%; Figure 3B). Since PRRSV L1 was first reported in China, L1C has been the dominant sublineage. Although the number of L1A sublineage has gradually increased in recent years, the proportion of L1C sublineage is still as high as 87.70% (Figure 3C).

### 3.6. RFLP Analysis

**Figure 4:**
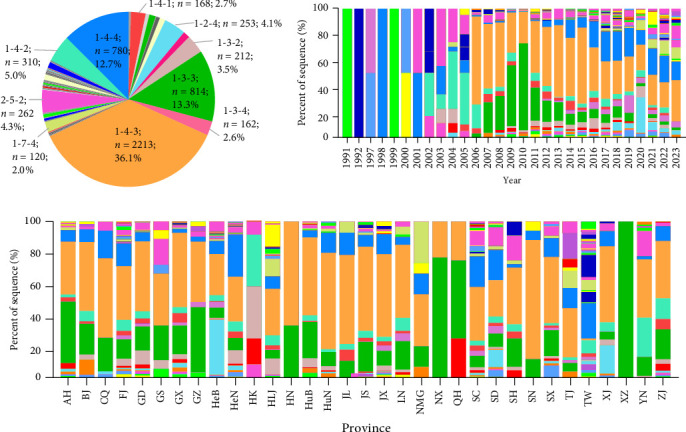
The restriction fragment length polymorphism (RFLP) pattern composition and spatiotemporal distribution of porcine reproductive and respiratory syndrome virus (PRRSV)-2 in China during 1991–2023. (A) The composition of RFLP pattern detected in China. Each color represents a different RFLP pattern, organized in ascending order for *Mlu* I, *Hinc* II, and *Sac* II enzyme patterns. (B) Temporal dynamics of PRRSV-2 RFLP patterns in China during 1991–2023. (C) Geographical distribution of PRRSV-2 RFLP patterns in China during 1991–2023.

Among the Chinese 6137 PRRSV-2 ORF5 sequences, 112 RFLP patterns were determined according to the previous PRRSV RFLP pattern method [13]. The three most common patterns, 1-4-3 (36.06%), 1-3-3 (13.26%), and 1-4-4 (12.71%), together constitute 62.03% of all PRRSV-2 sequences in China (Figure 4A). RFLP 1-4-3 detection ranged roughly from 19.58% to 68.37% during 2006–2023. RFLP 1-3-3 increased from 4.85% in 2006 to 67.97% in 2010, and then declined to 2.22% in 2023. RFLP 1-4-4, which represented less than 5% during 2006–2013, increased to 13.98% in 2014 and then dynamically changed in 11.20%~22.84% during 2015–2023 (Figure 4B). RFLP 1-4-3 was found in all provinces except Hong Kong and Tibet. Similarly, RFLP 1-3-3 was found in all provinces except Hong Kong and Taiwan. There are 72 RFLP patterns with less than five sequences; some RFLP patterns were detected only in one province, such as RFLP 1-65-2. Taiwan has the most RFLP patterns (*n* = 33), followed by Guangdong, Fujian, Shandong, and Henan province with the same number of RFLP patterns (*n* = 29; Figure 4C).

### 3.7. Comparison Between Lineage and RFLP of PRRSV-2

**Figure 5:**
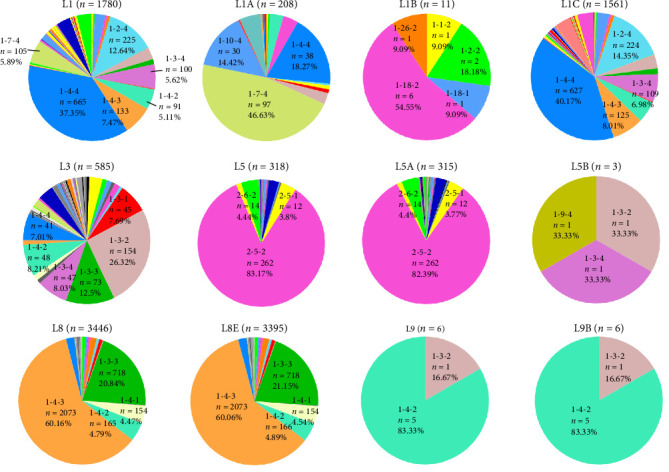
Detection frequency of restriction fragment length polymorphism (RFLP) patterns in porcine reproductive and respiratory syndrome virus (PRRSV)-2 open reading frame (ORF)-5–based lineages and sublineages in China during 1991–2023. The number and percentage of sequences belonging to the major RFLP patterns at lineage and sublineage level are shown.

Lineage classification and RFLP typing are two distinct methods for classifying PRRSV-2. The correlation between these two methods was analyzed. Some PRRSV-2 strains belong to same lineages, but they were classified into different RFLP patterns. For example, PRRSV-2 L1 has the most RFLP patterns (*n* = 56), followed by L3 (*n* = 44), L5 (*n* = 21), L8 (*n* = 39), and L9 (*n* = 2) (Figure 5). Notably, PRRSV-2 strains GZDJ and GZKB (GenBank No. EU140604 and EU140605, respectively) of the L8 lineage have 99.7% homology, but they were divided into different RFLP patterns (Supporting Information: Figure S1). Correspondingly, some PRRSV-2 strains belong to same RFLP patterns, but they were classified into different lineages. More than 26% RFLP patterns were detected in two or more lineages. For example, RFLP 1-3-2 pattern was detected in all lineages existed in China. In addition, RFLP 1-4-2, 1-3-4, 1-2-2 were detected in four lineages, of which RFLP 1-4-2 was one of the frequent patterns in L3 and L9 (Figure 5).

### 3.8. Geographic Distribution and Temporal Changes of RFLP 1-4-4 PRRSV

**Figure 6:**
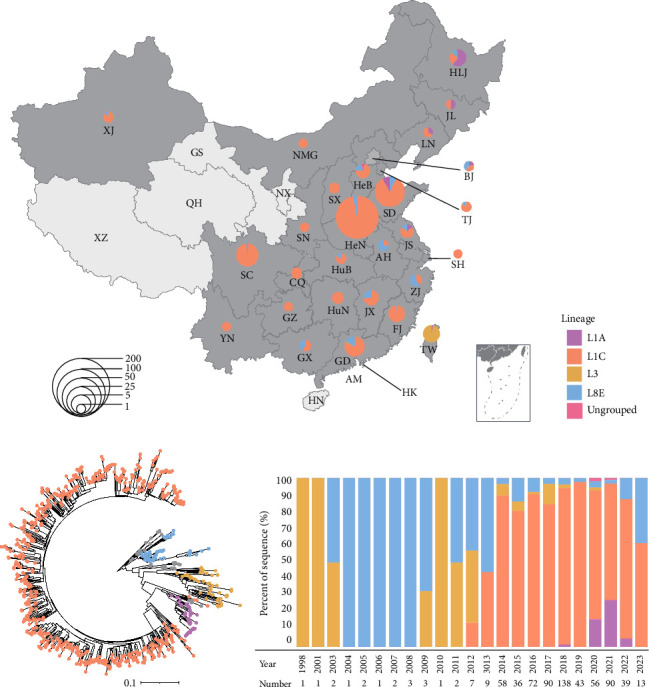
Lineage composition and spatiotemporal distribution of porcine reproductive and respiratory syndrome virus (PRRSV) restriction fragment length polymorphism (RFLP) 1-4-4 in China during 1991–2023. (A) Distribution of the sub-lineages in PRRSV-2 RFLP 1-4-4 pattern during 1991–2023. The number of strains and their relative proportions are illustrated by pie charts in each province. (B) Phylogenetic trees showing PRRSV-2 open reading frame (ORF)-5–based sublineages within RFLP 1-4-4 pattern. The PRRSV-2 ORF5 sequences with RFLP 1-4-4 pattern were classified into four different sublineages including L1A, L1C, L3, and L8E. (C) Temporal dynamics of PRRSV-2 sublineages in PRRSV-2 RFLP 1-4-4 pattern during 1991–2023.

During 1991–2023, a total of 780 RFLP 1-4-4 PRRSV strains were identified in China, representing 12.71% of all Chinese PRRSV-2 strains. For the geographical distribution, RFLP 1-4-4 PRRSVs were detected in 28 provinces, such as Henan (*n* = 155, 19.87%), Shandong (*n* = 98, 12.56%), and Sichuan (*n* = 64, 8.21%; Figure 6A). Among the 780 RFLP 1-4-4 PRRSVs, 778 strains were divided into four different lineages or sublineages, L1A, L1C, L3, and L8E, while the remaining two strains (GenBank No. OL546227 and OL546253) did not belong to any lineage. Most of the RFLP 1-4-4 PRRSVs belong to L1C (627/780, 80.38%), followed by L8E (72/780, 9.23%), L3 (41/780, 5.26%), and L1A (38/780, 4.87%; Figure 6B). Among them, RFLP 1-4-4 L1C sequences existed in 27 provinces. RFLP 1-4-4 L8E sequences existed in 17 provinces, but the number of sequences in each province was less than nine. RFLP 1-4-4 L1A mainly existed in the Northeastern region, including Heilongjiang (55.26%), Liaoning (2.63%), and Jilin (5.26%). For the temporal changes, the first RFLP 1-4-4 PRRSV was detected in China in 1998, which belonged to L3 lineage. RFLP 1-4-4 L1C PRRSVs were detected in 2012, rising from 44.44% of RFLP 1-4-4 PRRSVs in 2013 to over 90% in 2019 and declined to 60% during in 2023 (Figure 6C).

### 3.9. Lineage or Sublineages of Commercial PRRSV-2 Vaccines in China

Based on the ORF5 sequences, the currently main commercial PRRSV-2 vaccines in China were classified their lineages. The MLVs were VR-2332 (L5A), R98 (L5A), CH-1R (L8E), JXA1-R (L8E), HuN4-F112 (L8E), TJM-F92 (L8E), GDr180 (L8E), and PC (L8E). The inactivated vaccine CH-1a was L8E. To investigate the match between the vaccines and the circulating strains, the PRRSV-2 sequences (*n* = 729) in China from 2021 to 2023 were analyzed. The sequences were classified into seven sublineages, including L1A, L1B, L1C, L3, L5A, L8E, and L8-ungrouped. L1C strains have the largest number, accounting for 35.56%~46.91% during 2021–2023, followed by L8E (27.64%−37.78%) and L1A (15.46%−18.88%; Supporting Infomation: Figure S2).

## 4. Discussion

PRRS has caused significant economic losses to the swine industry since it was first reported in China in 1996 [19]. Due to high frequency of variation and recombination, the genetic diversity of PRRSV-2 was complex [20]. The epidemic of PRRSV-2 in China was previously divided into three stages. In 1996, PRRSV-2 was first reported in China, and the strains were regarded as classical PRRSVs, represented by CH-1a and BJ-4 strains [21, 22]. In 2006, a HP-PRRSV variant emerged, with the representative strains JXA1, HuN4, and JXwn06 [23–25]. In 2012, NADC30-like PRRSV was imported into China and gradually rise to dominant strain [26, 27]. Meanwhile, NADC34-like PRRSV belonging to L1 was also detected in China in 2017 [28, 29].

To better understand genetic diversity of PRRSV-2, the lineage classification system was updated from 9-lineages to 11-lineages classification system in 2023 [10]. The new lineage classification system is more detailed and standardized, avoiding the incomparability caused by differences in criteria among different researchers. In the present study, a dataset of all available 6137 PRRSV-2 ORF5 sequences in China was analyzed based on the 11-lineages classification system. As a result, the Chinese PRRSV-2 strains were classified into five lineages (L1, L3, L5, L8, and L9) and eight sublineages (L1A-L1C, L5A, L5B, L8C, L8E, and L9B). Compared with the previous PRRSV classification results in China, the sublineages classification is clearer than before. The lineage classification system facilitates tracking of emerging and endemic variants across time and space.

Furtherly, we analyzed the distribution characteristics of PRRSV-2 in China. L8 PRRSVs were detectable in all provinces in China except Taiwan. L3 PRRSVs were first detected in Taiwan [30], according for 91.07% (153/168) of PRRSV-2 strains in Taiwan, and now the L3 PRRSV-2 is circulating in South China, including Fujian, Guangdong, and Guangxi provinces. L5 PRRSV-2 in China was likely to be imported from the North America since the representative strain (BJ-4) shares 99.8% and 99.6% of genomic homology with RespPRRS MLV and VR-2332, respectively [31]. L1 was reported to be imported from North America before 2013 [32], and L1 PRRSV has become the dominant circulating PRRSV in China since 2017. Based on the epidemic of PRRSV-2 over the past three decades, it may predict that PRRSV-2 will primarily be dominated by the L1 lineage in China. Additionally, the L3 lineage is likely to further spread in the future.

RFLP typing was another method to describe the diversity of PRRSV-2, which has been widely adopted for veterinarians and farmers in the USA [10, 11]. Since the RFLP typing cannot capture PRRSV-2 genetic changes outside the cutting sites of the three restriction enzymes, it cannot accurately reflect genetic differences and relatedness of various PRRSV-2 strains, which means that viruses assigned to the same RFLP-type often are not closely related and vice versa. For example, some strains belonging to the same lineage, with more than 99.2% homology, were classified into different RFLP patterns. However, temporal and geographic distributions of different RFLP patterns still may reflect extensive PRRSV-2 genetic diversity. In the study, 6137 PRRSV-2 ORF5 sequences were divided into 112 RFLP patterns. There were only seven RFLP patterns before 2000, and there were 51 RFLP patterns in 2018. The increasing number of different RFLP patterns demonstrates the increasing genetic diversity of PRRSV-2 in China.

A high mortality and morbidity of PRRSV 1-4-4 L1C variant was emerged in the USA [33], which has attracted the attention of the world pig industry. In this study, 780 PRRSV strains with RFLP1-4-4 pattern was founded in China, those were divided into four sublineages, including L1A, L1C, L3, and L8E. However, up to now, no pandemic of RFLP 1-4-4 L1C variant has occurred in China. The L1C viruses have been further classified into five groups (L1C.1–L1C.5), and the RFLP 1-4-4 L1C variant in the USA was divided into L1C.5 sublineage according to the refined classification method [13], while there is no L1C.5 sublineage in China (data not shown). Given that China imports breeding pigs and frozen pork products from the USA, it is essential to strengthen the inspection and quarantine of imported breeding pigs and frozen pork products to prevent the spread of the RFLP 1-4-4 L1C variant.

In summary, to our knowledge, this study is the most comprehensive analysis of PRRSVs in China, and all the available PRRSV-2 in China were reclassified based on the new lineage classification criteria. The results revealed that the L1C has gradually replaced L8E to be a dominant epidemic strain, the L3 has a trend of spreading gradually from the southern to the northern provinces, and L1B PRRSV-2 in China was analyzed for the first time. The spatiotemporal distribution of different lineage or sublineage PRRSV-2 in China was analyzed too. Those findings contributed the understanding of genetic diversity and spatiotemporal distribution of PRRSV-2 in China and provide important references for future PRRSV-2 monitoring and control in China.

## Data Availability

The data that supports the findings of this study are available in the supporting information of this article.

## Supporting Information

Supporting Information 1 Figure S1. The percent identity between different RFLP patterns of the same lineage detected in China

Supporting Information 2 Figure S2. The temporal dynamics of PRRSV-2 sub-lineages in China during 2021–2023

Supporting Information 3 Table S1. Information of PRRSV-2 in China during 1991–2023

Supporting Information 4 Table S2. Homology analysis of GP5 amino acid sequences in PRRSV-2 of sub-lineages L1A-L1F and L1H-L1J

## References

[1] Han D., Hu Y., Li L. (2014). Highly Pathogenic Porcine Reproductive and Respiratory Syndrome Virus Infection Results in Acute Lung Injury of the Infected Pigs. *Veterinary Microbiology*.

[2] Benfield D. A., Nelson E., Collins J. E. (1992). Characterization of Swine Infertility and Respiratory Syndrome (SIRS) Virus (Isolate ATCC VR-2332). *Journal of Veterinary Diagnostic Investigation*.

[3] Brinton M. A., Gulyaeva A. A., Balasuriya U. B. R. (2021). ICTV Virus Taxonomy Profile: Arteriviridae 2021. *The Journal of General Virology*.

[4] Darwich L., Gimeno M., Sibila M. (2011). Genetic and Immunobiological Diversities of Porcine Reproductive and Respiratory Syndrome Genotype I Strains. *Veterinary Microbiology*.

[5] Wang X., Bai X., Wang Y. (2023). Pathogenicity Characterization of PRRSV-1 181187-2 Isolated in China. *Microbial Pathogenesis*.

[6] Johnson C. R., Griggs T. F., Gnanandarajah J., Murtaugh M. P. (2011). Novel Structural Protein in Porcine Reproductive and Respiratory Syndrome Virus Encoded by an Alternative ORF5 Present in All Arteriviruses. *The Journal of General Virology*.

[7] Key K. F., Haqshenas G., Guenette D. K., Swenson S. L., Toth T. E., Meng X.-J. (2001). Genetic Variation and Phylogenetic Analyses of the ORF5 Gene of Acute Porcine Reproductive and Respiratory Syndrome Virus Isolates. *Veterinary Microbiology*.

[8] Chen X. W., Li L., Yin M. (2017). Cloning and Molecular Characterization of the ORF5 Gene from a PRRSV-SN Strain from Southwest China. *Microbial Pathogenesis*.

[9] Jakab S., Kaszab E., Marton S. (2022). Genetic Diversity of Imported PRRSV-2 Strains, 2005-2020, Hungary. *Frontiers in Veterinary Science*.

[10] Wesley R. D., Mengeling W. L., Lager K. M., Clouser D. F., Landgraf J. G., Frey M. L. (1998). Differentiation of a Porcine Reproductive and Respiratory Syndrome Virus Vaccine Strain from North American Field Strains by Restriction Fragment Length Polymorphism Analysis of ORF 5. *Journal of Veterinary Diagnostic Investigation*.

[11] Trevisan G., Sharma A., Gauger P. (2021). PRRSV2 Genetic Diversity Defined by RFLP Patterns in the United States from 2007 to 2019. *Journal of Veterinary Diagnostic Investigation*.

[12] Cha S. H., Chang C. C., Yoon K. J. (2004). Instability of the Restriction Fragment Length Polymorphism Pattern of Open Reading frame 5 of Porcine Reproductive and Respiratory Syndrome Virus during Sequential Pig-to-Pig Passages. *Journal of Clinical Microbiology*.

[13] Yim-Im W., Anderson T. K., Paploski I. A. D. (2023). Refining PRRSV-2 Genetic Classification Based on Global ORF5 Sequences and Investigation of Their Geographic Distributions and Temporal Changes. *Microbiology Spectrum*.

[14] Shi M., Lam T. T., Hon C. C. (2010). Phylogeny-Based Evolutionary, Demographical, and Geographical Dissection of North American type 2 Porcine Reproductive and Respiratory Syndrome Viruses. *Journal of Virology*.

[15] Li J. H., Xu H., Li C. (2024). Genomic Characterization of HLJDZD55: The First L1B PRRSV in China. *Transboundary and Emerging Diseases*.

[16] Li C., Zhuang J., Wang J. (2016). Outbreak Investigation of NADC30-Like PRRSV in South-East China. *Transboundary and Emerging Diseases*.

[17] Gao J. C., Xiong J. Y., Ye C. (2017). Genotypic and Geographical Distribution of Porcine Reproductive and Respiratory Syndrome Viruses in Mainland China in 1996-–2016. *Veterinary Microbiology*.

[18] Nguyen L.-T., Schmidt H. A., von Haeseler A., Minh B. Q. (2015). IQ-TREE: A Fast and Effective Stochastic Algorithm for Estimating Maximum-Likelihood Phylogenies. *Molecular Biology and Evolution*.

[19] Firth A. E., Zevenhoven-Dobbe J. C., Wills N. M. (2011). Discovery of a Small Arterivirus Gene that Overlaps the GP5 Coding Sequence and Is Important for Virus Production. *The Journal of General Virology*.

[20] Li C., Zhao J., Li W. (2024). Prevalence and Genetic Evolution of Porcine Reproductive and Respiratory Syndrome Virus in Commercial Fattening Pig Farms in China. *Porcine Health Management*.

[21] An T. Q., Zhou Y. J., Liu G. Q. (2007). Genetic Diversity and Phylogenetic Analysis of Glycoprotein 5 of PRRSV Isolates in Mainland China from 1996 to 2006: Coexistence of Two NA-Subgenotypes with Great Diversity. *Veterinary Microbiology*.

[22] Ran Z. G., Chen X. Y., Yang H. C., Guo X., Gai X. N. (2007). Recovery of an Infectious Virus from the Full-Length cDNA of PRRSV BJ-4. *Acta Microbiologica Sinica*.

[23] Han W., Wu J. J., Deng X. Y. (2009). Molecular Mutations Associated With the in Vitro Passage of Virulent Porcine Reproductive and Respiratory Syndrome Virus. *Virus Genes*.

[24] Leng X., Li Z., Xia M. (2012). Mutations in the Genome of the Highly Pathogenic Porcine Reproductive and Respiratory Syndrome Virus Potentially Related to Attenuation. *Veterinary Microbiology*.

[25] Tian Z. J., An T. Q., Zhou Y. J. (2009). An Attenuated Live Vaccine Based on Highly Pathogenic Porcine Reproductive and Respiratory Syndrome Virus (HP-PRRSV) Protects Piglets Against HP-PRRS. *Veterinary Microbiology*.

[26] Zhou L., Wang Z., Ding Y., Ge X., Guo X., Yang H. (2015). NADC30-Like Strain of Porcine Reproductive and Respiratory Syndrome Virus, China. *Emerging Infectious Diseases*.

[27] Wu Z., Chang T., Wang D. (2024). Genomic Surveillance and Evolutionary Dynamics of Type 2 Porcine Reproductive and Respiratory Syndrome Virus in China Spanning the African Swine Fever Outbreak. *Virus Evolution*.

[28] Zhang H. L., Zhang W. L., Xiang L. R. (2018). Emergence of Novel Porcine Reproductive and Respiratory Syndrome Viruses (ORF5 RFLP 1-7-4 Viruses) in China. *Veterinary Microbiology*.

[29] Xu H., Song S., Zhao J. (2020). A Potential Endemic Strain in China: NADC34-Like Porcine Reproductive and Respiratory Syndrome Virus. *Transboundary and Emerging Diseases*.

[30] Zhang W. L., Zhang H. L., Xu H. (2019). Two Novel Recombinant Porcine Reproductive and Respiratory Syndrome Viruses Belong to Sublineage 3.5 Originating from Sublineage 3.2. *Transboundary and Emerging Diseases*.

[31] Guo Z., Chen X.-X., Li R., Qiao S., Zhang G. (2018). The Prevalent Status and Genetic Diversity of Porcine Reproductive and Respiratory Syndrome Virus in China: A Molecular Epidemiological Perspective. *Virology Journal*.

[32] Zhao K., Ye C., Chang X. B. (2015). Importation and Recombination Are Responsible for the Latest Emergence of Highly Pathogenic Porcine Reproductive and Respiratory Syndrome Virus in China. *Journal of Virology*.

[33] Trevisan G., Li G., Moura C. A. A. (2021). Complete Coding Genome Sequence of a Novel Porcine Reproductive and Respiratory Syndrome Virus 2 Restriction Fragment Length Polymorphism 1-4-4 Lineage 1C Variant Identified in Iowa, USA. *Microbiology Resource Announcements*.

