# Supplementary figures and images for: Refining Lineage Classification and Updated RFLP Patterns of PRRSV-2 Revealed Viral Spatiotemporal Distribution Characteristics in China in 1991–2023
Source: Transbound Emerg Dis. 2025 Mar 9;2025:9977088. doi: 10.1155/tbed/9977088 (PMC12017074; doi:10.1155/tbed/9977088)

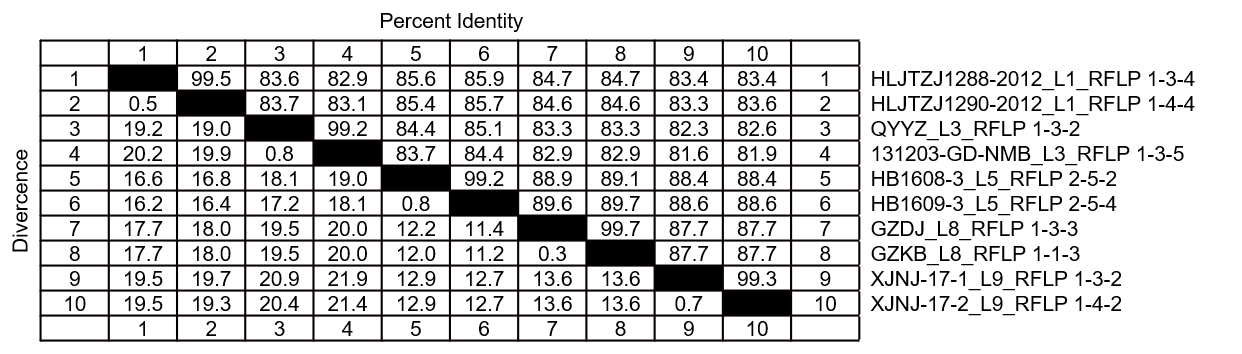

Supplement: Supporting Information 1 — Figure S1. The percent identity between different RFLP patterns of the same lineage detected in China [file 9977088.f1.tif]

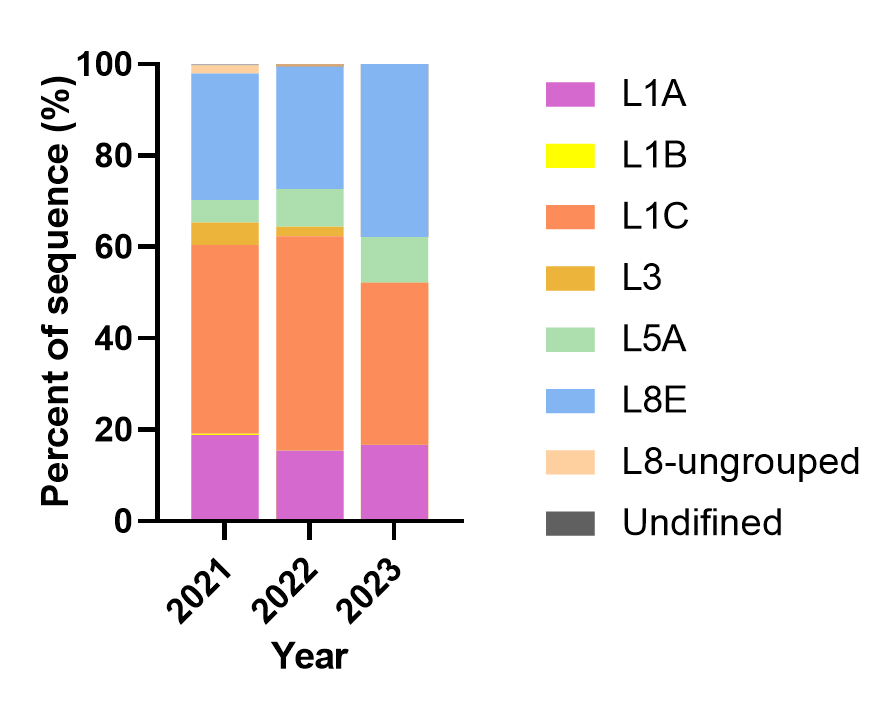

Supplement: Supporting Information 2 — Figure S2. The temporal dynamics of PRRSV-2 sub-lineages in China during 2021–2023 [file 9977088.f2.tif]
