# Supplementary material for: Refining Lineage Classification and Updated RFLP Patterns of PRRSV-2 Revealed Viral Spatiotemporal Distribution Characteristics in China in 1991–2023
Source: Transbound Emerg Dis. 2025 Mar 9;2025:9977088. doi: 10.1155/tbed/9977088 (PMC12017074; doi:10.1155/tbed/9977088)
Supplement: Supporting Information 4 — Table S2. Homology analysis of GP5 amino acid sequences in PRRSV-2 of sub-lineages L1A-L1F and L1H-L1J [file 9977088.f4.docx]

Table S2 Homology analysis of GP5 amino acid sequences in PRRSV-2 of sublineages L1A-L1F and L1H-L1J

| Lineage | Homology analysis of GP5 amino acid sequences in L1 sublineages (%) | | | | | | | |  |
| --- | --- | --- | --- | --- | --- | --- | --- | --- | --- |
|  | L1A | L1B | L1C | L1D | L1E | L1F | L1H | L1I | L1J |
| L1A | 97.0-100 |  |  |  |  |  |  |  |  |
| L1B | 91.5-96.0 | 94.5-98.0 |  |  |  |  |  |  |  |
| L1C | 88.1-92.0 | 87.1-93.5 | 88.6-95.5 |  |  |  |  |  |  |
| L1D | 89.1-94.0 | 88.6-94.5 | 86.6-95.5 | 92.0-97.0 |  |  |  |  |  |
| L1E | 86.6-89.6 | 86.6-91.0 | 85.1-89.1 | 85.1-89.1 | 95.5-99.0 |  |  |  |  |
| L1F | 90.5-94.0 | 87.6-91.0 | 84.6-94.0 | 87.1-94.5 | 84.1-89.1 | 96.0-97.5 |  |  |  |
| L1H | 90.0-93.5 | 89.1-93.0 | 85.1-94.0 | 89.6-97.0 | 83.1-87.1 | 88.6-93.5 | 95.0-100 |  |  |
| L1I | 88.1-90.5 | 87.6-91.0 | 84.6-89.1 | 88.1-91.5 | 84.6-87.6 | 86.6-90.0 | 87.1-89.6 | 95.0-100 |  |
| L1J | 90.0-94.0 | 89.1-92.5 | 85.6-94.0 | 88.6-94.0 | 85.1-88.1 | 87.6-93.5 | 87.1-93.0 | 86.6-90.0 | 90.0-96.5 |
